# Reconsolidation of Motor Memories Is a Time-Dependent Process

**DOI:** 10.3389/fnhum.2016.00408

**Published:** 2016-08-17

**Authors:** Toon T. de Beukelaar, Daniel G. Woolley, Kaat Alaerts, Stephan P. Swinnen, Nicole Wenderoth

**Affiliations:** ^1^Movement Control and Neuroplasticity Research Group, Department of Kinesiology, Katholieke Universiteit (KU) LeuvenLeuven, Belgium; ^2^Neural Control of Movement Group, Department of Health Sciences and Technology, ETH ZürichZurich, Switzerland; ^3^Research Group for Neuromotor Rehabilitation, Department of Rehabilitation Sciences, Katholieke Universiteit (KU) LeuvenLeuven, Belgium

**Keywords:** consolidation, reconsolidation, motor learning, sequence task, memory updating

## Abstract

Reconsolidation is observed when a consolidated stable memory is recalled, which renders it transiently labile and requires re-stabilization. Motor memory reconsolidation has previously been demonstrated using a three-day design: on day 1 the memory is encoded, on day 2 it is reactivated and experimentally manipulated, and on day 3 memory strength is tested. The aim of the current study is to determine specific boundary conditions in order to consistently degrade motor memory through reconsolidation paradigms. We investigated a sequence tapping task (*n* = 48) with the typical three-day design and confirmed that reactivating the motor sequence briefly (10 times tapping the learned motor sequence) destabilizes the memory trace and makes it susceptible to behavioral interference. By systematically varying the time delay between memory reactivation and interference while keeping all other aspect constant we found that a short delay (i.e., 20 s) significantly decreased performance on day 3, whereas performance was maintained or small (but not significant) improvements were observed for longer delays (i.e., 60 s). We also tested a statistical model that assumed a linear effect of the different time delays (0 s, 20 s, 40 s, 60 s) on the performance changes from day 2 to day 3. This linear model revealed a significant effect consistent with the interpretation that increasing time delays caused a gradual change from performance degradation to performance conservation across groups. These findings indicate that re-stabilizing motor sequence memories during reconsolidation does not solely rely on additional motor practice but occurs with the passage of time. This study provides further support for the hypothesis that reconsolidation is a time-dependent process with a transition phase from destabilization to re-stabilization.

## Introduction

Acquiring a novel task leads to a new but initially fragile memory (Duncan, 1949; Misanin et al., 1968; Dudai, 1996; McGaugh, 2000). This initial memory is highly susceptible to interference and is in need of consolidation. The process of consolidation makes the memory more robust and resistant against competing influences and stabilizes memory representations despite the absence of any further training (Brashers-Krug et al., 1996; Walker et al., 2002, 2003; Korman et al., 2003, 2007; Censor and Cohen, 2011). Consolidation has been demonstrated for several memory domains including motor memories that are formed when a new task is practiced repetitively (Brashers-Krug et al., 1996; Walker et al., 2002, 2003). Hallmark features of this process are an increase in motor performance (often estimated by a shift in the speed-accuracy function, i.e., movements are performed faster and more accurately after training) and a decrease in motor variability (Reis et al., 2009; Shmuelof et al., 2012). One motor task often used for studying motor memory consolidation is sequence tapping. A short sequence of 5–8 elements (each representing one tap with a specific finger) is usually performed either by typing the sequence on a keyboard (Walker et al., 2002, 2003) or as a finger-to-thumb opposition task (Karni et al., 1998). Practicing this task triggers a process in which multiple elements of the movement are integrated into one single behavior which is typically reflected by an increase in both tapping accuracy (i.e., producing the correct sequence) and in speed (Walker et al., 2002, 2003). Previous research investigating motor memory consolidation used this task and showed that practicing a novel sequence over twelve 30 s trials results in significant performance gains which reached plateau at the end of training (Walker et al., 2002, 2003). Further increases in performance can be observed once the memory is consolidated and large “offline gains” have been consistently observed after one night of sleep (i.e., performance increases significantly relative to the plateau performance reached at the end of training; Fischer et al., 2002; Walker et al., 2002, 2003, 2005; Stickgold, 2005).

Considerable evidence indicates that consolidation is a time-dependent process, with memories only susceptible to enhancement or disruption when specific interventions are provided shortly (i.e., within hours) after initial memory encoding, nevertheless not once this critical time-window has closed (Davis and Squire, 1984; Brashers-Krug et al., 1996; McGaugh, 2000; Walker et al., 2002, 2003). These findings have led to the long-held view that once a memory is truly consolidated it is rigid and can no longer be modulated. However, when performing introspective analyses of personal memories it becomes apparent that memories are often not constant or rigid in terms of strength or content (Lee, 2009). Experimentally it has been shown that a consolidated memory can be disrupted when an amnesic agent is presented shortly after memory retrieval. This effect was not observed when the administration of the amnesic agent was not preceded by retrieval, or when retrieval was not followed by the amnesic agent (Misanin et al., 1968). This finding suggested that memory retrieval renders a seemingly consolidated memory fragile again and is in need of re-stabilization, a process known as reconsolidation. Nader et al. (2000) provided the first conclusive evidence that memory erasure can be caused by interference during reconsolidation. Particularly, they showed in rodents that a conditioned fear memory can be blocked by injecting a protein synthesis inhibitor (a “consolidation blocker”) immediately after reactivation. These findings caused a rapid increase in animal research investigating the process of memory *reconsolidation* in further detail (Nader and Einarsson, 2010; Besnard et al., 2012).

Reconsolidation has been investigated in several memory domains in humans (for review see Schiller and Phelps, 2011) including the motor memory domain (Walker et al., 2003; Censor et al., 2010, 2014; Hardwicke et al., 2016). To do so, most studies used a three-day design and applied an interference approach involving: (i) acquisition of a new motor task A on day 1; (ii) reactivation and experimental manipulation of motor task A on day 2; and finally; (iii) assessment of potential changes in memory strength of motor task A on day 3 (Walker et al., 2003; Censor et al., 2010, 2014; Hardwicke et al., 2016). A seminal study by Walker et al. (2003) used this three-day design and showed that when motor sequence A was learned on day 1 but then physically reactivated and subjected to experimental interference on day 2 (by practicing a new sequence B immediately afterwards), the accuracy of sequence A on day 3 decreased significantly relative to that on day 2, indicating true memory degradation. Importantly, no such memory degradation was observed when reactivation of sequence A was not followed by training of the interfering sequence B, or when sequence B was trained without prior reactivation of sequence A (Walker et al., 2003). However, later studies reported difficulties in replicating the finding that memory can be degraded during reconsolidation even when the identical task design and protocol were used as in Walker et al. (2003), de Beukelaar et al. (2014) and Hardwicke et al. (2016). Other studies used non-invasive brain stimulation to interfere with memory formation in primary motor cortex (M1; Censor et al., 2010, 2014) and found that applying repetitive transcranial magnetic stimulation (1 Hz rTMS) over M1 on day 2 immediately after reactivation of sequence A did not cause performance to drop on day 3. It did, however, block further gains in performance typically observed after a night of sleep between day 2 and day 3.

These divergent results reflect an ongoing scientific debate concerning the functional role of reconsolidation in the modification of stored memories and gave rise to two competing hypotheses (Lee, 2009): first, the “destabilization theory” posits that in order to modify a memory it needs to be destabilized so that new information can be added. Subsequently the modified memory is “re-stabilized” in order to generate an improved memory trace for future recall. Importantly, this hypothesis predicts that causing interference during the destabilization phase results in memory loss. This concept is consistent with most animal work (Besnard et al., 2012) and several human studies showing that interference after reactivation can lead to significant deterioration of task performance when probed during a retention test (Walker et al., 2003; Kindt et al., 2009; Chan and LaPaglia, 2013). The “updating theory” on the other hand, postulates that reactivating a stable memory may indeed open a time-window for memory modification, but importantly, there is no initial destabilization phase. Several human studies support this notion, showing that interference only blocks performance gains that one would normally observe when memory formation is uninterrupted, but that the interference could not induce performance decrements (Rodriguez-Ortiz and Bermúdez-Rattoni, 2007; Censor et al., 2010; Hardwicke et al., 2016).

When comparing divergent results between human and animal work, it should be noted that in humans, memory interference is mostly induced using methods which target the neural basis of the memory in an anatomically and mechanistically unspecific manner, e.g., by acquiring a competing task (Walker et al., 2003; Forcato et al., 2007; Hupbach et al., 2007; Chan and LaPaglia, 2013; de Beukelaar et al., 2014; Hardwicke et al., 2016), by orally administered drugs like propranolol (Brunet et al., 2008; Kindt et al., 2009; Soeter and Kindt, 2011) or by applying invasive (Kroes et al., 2014) and non-invasive brain stimulation (Censor et al., 2010). In animal work on the other hand, methods are being used that directly target the molecular underpinnings of memory formation, e.g., by injecting consolidation inhibiting proteins directly into the brain areas responsible for memory formation (Nader et al., 2000). Other factors might also contribute to divergent results, such as subtle boundary conditions that may constrain the extent to which a memory can be experimentally interfered with upon reactivation. For example, in animal research it has been shown that specific determinants should be precisely controlled, such as the age of the memory (Milekic and Alberini, 2002; Suzuki et al., 2004), intensity of training (Eisenberg et al., 2003; Suzuki et al., 2004; Wang et al., 2009), reactivation length (Eisenberg et al., 2003; Pedreira and Maldonado, 2003; Suzuki et al., 2004), and novelty of information provided during the reactivation session (Pedreira et al., 2004; Morris et al., 2006; Díaz-Mataix et al., 2013). In humans, however, these boundary conditions are currently not well understood (Schiller and Phelps, 2011; Auber et al., 2013; Sevenster et al., 2013; Sandrini et al., 2015).

In a previous study, we showed that the length of reactivation on day 2 (i.e., actively performing sequence A) is a crucial boundary condition to effectively show a degradation of the motor memory after interfering with the induced reconsolidation process (de Beukelaar et al., 2014). A clear relationship between the length of reactivation and motor memory degradation was found, indicating that the longer the reactivation phase, the minimal the decline in performance due to interference when retested 24 h later. However, it remains unclear whether the re-stabilization observed during prolonged reactivation (i.e., tapping sequence A for more than 60 s) is triggered by continuous physical practice, or whether re-stabilization would also occur automatically with the passage of time after a short reactivation. Here we test the hypothesis that increasing the delay between a standardized short reactivation and an interfering intervention reduces memory degradation when tested the next day, suggesting that even though reconsolidation destabilizes the memory initially, this state is maintained only for a limited time-window.

## Materials and Methods

### Subjects

Forty-eight right-handed subjects (*n* = 12 per group; 17 men; mean age 23.1; range 18–32 years) volunteered for this study. None were practiced musicians nor had extensive gaming experience, as assessed by a self-report questionnaire. All subjects were naïve to the purpose of the study and gave written informed consent prior to participation. Experimental procedures were approved by the local Ethics Committee for Biomedical Research at Katholieke Universiteit (KU) Leuven and conformed to the Declaration of Helsinki.

Subjects were instructed to sleep for a minimum of 6 h per night prior to and after the experimental sessions to avoid general fatigue and ensure overnight consolidation. Subjects were instructed not to take daytime naps or consume alcohol, and not to practice motor sequences in between sessions.

### Motor Task

**Figure 1:**
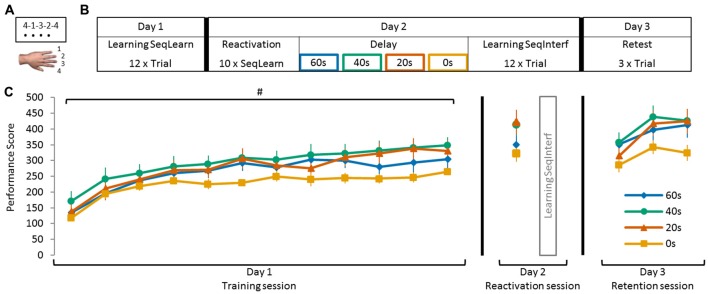
**Schematic representation of the sequence tapping task and experimental protocol. (A)** The sequence tapping task was performed with the left non-dominant hand on a laptop keyboard. Two different 5-element sequences were used; a learning (SeqLearn) and an interference sequence (SeqInterf) respectively. An experimental trial consisted of 30 s of sequence tapping followed by a rest period of 30 s to prevent fatigue. Participants were instructed to type the sequences as quickly and as accurately as possible. **(B)** The experiment was conducted on three consecutive days. On day 1 (*training session*) subjects were trained on one sequence (SeqLearn) for 12 trials of 30 s. On day 2, the motor memory was reactivated by tapping SeqLearn 10 times (i.e., 50 key presses; irrespective of whether they were correct or not; *reactivation session*) and was followed by learning a new sequence (SeqInterf). Subjects were randomly assigned to groups with a different delay between the reactivation and the interference sequence, which were either 0 s, 20 s, 40 s or 60 s. On day 3 of the experiment (*retention session*), final performance levels on the SeqLearn (3 × 30 s) and SeqInterf (3 × 30 s) were measured. **(C)** Visualization of the performance data of all experimental groups (0 s, 20 s, 40 s, and 60 s) presented in the temporal order of the testing protocol. Performances are shown as collected in the separate sessions; on day 1 and day 3 subjects performed trials of 30 s (shown as PerfScore_all_) while on day 2 they briefly tapped SeqLearn 10 times (shown as PerfScore_10_). Significant main *trial* effect is indicated by ^#^(*p* < 0.001). Vertical bars indicate SEs.

Subjects were comfortably seated in front of a laptop in a quiet room free of visual distractions. Motor memory formation was probed with a sequence tapping task, adapted from Karni et al. (1998), that has been used previously in motor reconsolidation research (Walker et al., 2003; Censor et al., 2010, 2014; de Beukelaar et al., 2014). Participants performed the sequence tapping task with their left (non-dominant) hand to reduce the likelihood of a ceiling effect during learning. Key presses were recorded by four neighboring keys labeled 1, 2, 3 and 4, which corresponded to the little, ring, middle and index finger, respectively (Figure 1A). Two different 5-element sequences (A: 4-1-3-2-4 and B: 2-3-1-4-2) were used interchangeably throughout the experiment; one being the learning sequence (*SeqLearn*) and the other the interfering sequence (*SeqInterf*). Sequences were randomized and counter-balanced across subjects.

Subjects initiated the behavioral task themselves by pressing the spacebar key on the laptop. The required sequence was then shown on top of the screen (each number represented a finger tap as specified above). While performing the task, each key press produced a black dot underneath the number indicating which finger should have been used. Note that this feedback indicated only that a key press was registered, but not whether the correct key had been selected (Figure 1A). Once a sequence was completed (i.e., when 5 keys were pressed irrespective of whether they were correct or not) the screen was refreshed so that all black dots were removed, while the sequence of numbers remained visible. An experimental trial consisted of 30 s sequence tapping followed by 30 s of rest to prevent fatigue. During the rest period the screen turned white. The trials following the rest period started automatically and subjects were continuously motivated throughout the experiment to type the sequences as quickly and accurately as possible.

### Protocol

For each subject the experiment was conducted at the same time of the day on three consecutive days to account for possible circadian rhythm effects. During the first day of the experiment (*training session*) subjects practiced the sequence for 12 trials (SeqLearn). On the second day of the experiment (*reactivation session*) subjects reactivated SeqLearn by tapping the sequence a total of 10 times (irrespective of whether they were correct or incorrect) and were motivated to do this as quickly and as accurately as possible. Reactivation was followed by the acquisition of a new interfering sequence for 12 trials (SeqInterf). Subjects were instructed before reactivation that a new sequence had to be learned after reactivation, however, they did not know which sequence this would be. On the third and final day (*retention session*) subjects performed three SeqLearn trials, which were followed by three SeqInterf trials, to provide an indication of the final level of performance (Figures 1B,C).

Subjects were randomly allocated to one of the four experimental groups: in the first experimental group, reactivation was immediately followed by the acquisition of SeqInterf so that virtually no delay was present (0 s group). In the other experimental groups, the delay between reactivation and interference was 20 s, 40 s or 60 s (Figures 1B,C). Importantly, on day 2 all subjects received the identical instruction that they first had to tap SeqLearn, and subsequently learn a new sequence (SeqInterf) which would commence after the subject pressed the space bar. We did not inform the subject regarding the length of reactivation or the delay between reactivation and interference to minimize pacing strategies or other cognitive confounds. In the 0 s group the experimenter instructed subjects to press the space bar immediately after the 10 SeqLearn reactivation trials were completed. In the other groups, the delay (20 s, 40 s, and 60 s) was accurately timed by the experimenter and subjects were instructed on when to press the space bar. These delays were based on previous research showing that destabilization due to physical reactivation of SeqLearn only occurs for a duration <60 s of physical tapping (de Beukelaar et al., 2014). Accordingly, we chose 60 s as the maximum time interval in the present study even though we would expect analogous or even stronger re-stabilization effects for longer intervals. In summary, the experimental groups only differed with respect to the reactivation session on the second day. Specifically, the delay between reactivating SeqLearn and acquiring SeqInterf varied between 0 s and 60 s.

### Data Analysis and Statistics

Subjects performed the sequence tapping task on a laptop where the key presses were registered by a custom data collection program (E-Prime Psychology Software Tools, Inc., Shapsburg, PA, USA). Performance measures consisted of both accuracy and speed. Accuracy was calculated as the percentage of correct sequences (i.e., sequences where all key presses corresponded to the temporal order of the elements) relative to the total number of sequences tapped per 30 s trial (i.e., number of sequences tapped within 30 s irrespective of whether the order was correct or incorrect). Speed was measured as the time between key presses (in s), i.e., the inter-tap interval (ITI). Based on the “speed-accuracy trade off”, which indicates that for a given skill level accuracy is diminished when speed is increased, skill improvement is reflected by a shift in the speed-accuracy function (Reis et al., 2009; Shmuelof et al., 2012). de Beukelaar et al. (2014) reported a linear relationship between the accuracy percentage and ITI (*R* = 0.94). Therefore an overall *performance score* (PerfScore) was calculated for each subject and trial by dividing the percentage of accurately typed sequences by the ITI. A higher score indicates improved performance.

First we tested whether SeqLearn was acquired in a similar manner across groups on day 1. To do so, performance scores were calculated for the full 30 s tapping period (PerfScore_all_) and an analysis of variance (ANOVA) model was conducted with the within subjects factor *trial* (1–12) and the between subjects factor *group* (0 s; 20 s; 40 s; and 60 s). Additionally we tested whether the initial PerfScore_all_ measured during the first trial on day 1 was similar across groups using an ANOVA with the factor *group* (0 s; 20 s; 40 s; and 60 s). Furthermore, we tested whether a plateau was reached at the end of training using an ANOVA with the factors *group* and *trial* (10–12).

Next we tested overnight performance changes of SeqLearn from day 1 to day 2 and from day 2 to day 3 to investigate consolidation and reconsolidation processes, respectively. Since reactivation on day 2 required subjects to tap only 10 sequences we calculated the performance score only for the first 10 sequences tapped within a given 30 s trial (PerfScore_10_), thus increasing consistency of data analyses across the 3 days and minimizing confounds caused e.g., by fatigue (Brawn et al., 2010).

We first investigate performance changes due to offline consolidation between the end of training on day 1 and the reactivation on day 2. We calculated the *baseline* performance on day 1 as the mean PerfScore_10_ of trials 10–12 (note that the last 3 trials were chosen to have a more reliable estimate of the baseline performance on day 1). We then tested offline learning from day 1 to day 2 in all four experimental groups with an ANOVA analysis including the within subjects factor *day* (day 1, day 2) and the between subjects factor *group* (0 s; 20 s; 40 s; and 60 s).

**Figure 2:**
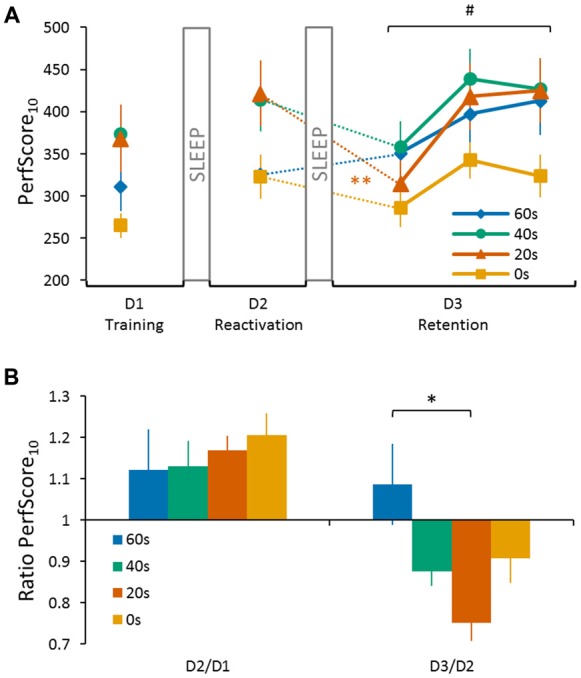
**Performance of the four experimental groups (0 s, 20 s, 40 s and 60 s) on the sequence tapping task over three consecutive days. (A)** PerfScore_10_ on day 1 (D1 Training) represents the average performance score (accuracy (%)/inter-tap interval (ITI; s)) of the first 10 tapped sequences (i.e., SeqLearn, irrespective of whether they were correct or incorrect) of the last three training trials. PerfScore_10_ on day 2 (D2 Reactivation) represents the reactivation performance obtained from tapping SeqLearn 10 times. PerfScore_10_ data on the third and final day (D3 Retention) represent the first 10 complete SeqLearn sequences of each trial. We found a significant *day* × *group* interaction (*F*_(3,44)_ = 5.28, *p* < 0.01) and a Tukey HSD *post hoc* analysis showed a significant drop in performance from day 2 to day 3 for the 20 s group (*p* < 0.01). The performance was only degraded during the first trial on day 3 and increased quickly during the subsequent two tapping trials (main *trial* effect *F*_(2,88)_ = 28.31, *p* < 0.001). **(B)** Performance ratios visualizing changes in performance between consecutive days. Performance ratios from day 1 to day 2 represent the change in the reactivation performance on day 2 (10 × SeqLearn) relative to the baseline performance level on day 1 (average of the first 10 tapped sequences of the last 3 training trials; D2/D1). Performance ratios from day 2 to day 3 represent the change in performance on day 3 (first 10 × SeqLearn) relative to reactivation performance on day 2 (10 × SeqLearn; D3/D2). A ratio <1 indicates memory loss while a ratio >1 indicates further memory improvement overnight, i.e., offline gains. We found a significant *group* main effect for the D3/D2 ratio (preplanned comparison with D2/D1 as a covariate of no interest; *F*_(3,40)_ = 3.71, *p* = 0.03, one-sided). A Tukey HSD *post hoc* analysis revealed a significant difference in performance from day 2 to day 3 for the 20 s group compared to the 60 s group (*p* < 0.01). Significant main *trial* effect is indicated by ^#^(*p* < 0.001). Significant Tukey HSD *post hoc* is indicated for the main *group* effect by *(*p* < 0.01), and the *day* × *group* interaction by **(*p* < 0.01). Vertical bars indicate SEs.

The reconsolidation effect was central to our research question and we performed an ANOVA analysis to specifically test whether the duration of the delay between reactivation and interference on day 2 has an influence on the extent of motor memory degradation on day 3. To do so, we conducted a repeated measures ANOVA on PerfScore_10_ for the within subjects factor *day* (day 2, day 3) and the between subjects factor *group* (0 s; 20 s; 40 s; and 60 s). Note that we considered only the first trial on day 3 because our previous study has indicated that memory degradation due to reconsolidation can only be temporarily observed and is quickly compensated when additional training is provided (de Beukelaar et al., 2014). To visualize performance changes between two consecutive days, a ratio was calculated by dividing the PerfScore_10_ of the latter by the former (i.e., D2/D1 and D3/D2). A ratio <1 indicates memory loss while a ratio >1 indicates further memory improvement overnight, i.e., offline gains (see Figure 2B).

One general concern is that individual differences in offline gains measured from day 1 to day 2 (note that all subjects have followed the same protocol up to this point) might have influenced performance changes measured from day 2 to day 3. In other words, larger offline gains from day 1 to day 2 might be followed by smaller gains from day 2 to day 3 consistent with the observation that learning curves follow a power-law. To consider this potential confound in our analysis, we first submitted the D3/D2 ratios and the D2/D1 ratios of each individual to a Pearson correlation analysis and estimated the strength of this potential association. Then, we performed a control analysis to ensure that the D3/D2 ratios differed across groups even if individual differences in D2/D1 offline gains are considered. To do so we submitted D3/D2 ratios to a general linear model with the between subject factor *group* (0 s; 20 s; 40 s; and 60 s) and included the D2/D1 ratios as a covariate of no interest. Based on our previous study we test the *a priori* hypothesis that there is a linear relationship between the different delay durations and performance changes from day 2 to day 3, more specifically shorter delays cause stronger memory degradation (i.e., no performance gains) than do longer delays between reactivation and interference. We tested the hypothesis directly via a preplanned comparison using the following contrast vector [−3, −1, 1, 3] for the 0 s, 20 s, 40 s and 60 s groups, thus modeling that performance gains at D3 compared to D2 increase linearly with the length of the delay (since we have strong prior evidence to expect a linear *increase* we report one-sided statistics for this comparison).

Finally, we tested whether the different delays between reactivating *SeqLearn* and acquiring the interfering sequence *SeqInterf* influenced PerfScore_10_ of the 3 tapping trials on day 3. An ANOVA model was conducted on the day 3 retention data with the between subjects factor *group* (0 s; 20 s; 40 s; and 60 s) and the within subjects factor *trial* (1–3).

Analogous analyses were performed for SeqInterf (see Figure 5).

The alpha level for all statistical tests was set to 0.05. *Post hoc* comparisons were performed with Tukey’s HSD test.

## Results

Four experimental groups of subjects practiced the sequence tapping task and initial performance (i.e., performance on the first 30 s trail on day 1) did not differ between the groups (no main *group* effect *F*_(3,44)_ = 1.11, *p* = 0.35). Furthermore, all experimental groups significantly improved PerfScore_all_ for SeqLearn over the course of training on day 1 (*trial* main effect *F*_(11,484)_ = 68.91, *p* < 0.001) and all groups exhibited similar learning gains (no *group* main effect *F*_(3,44)_ = 1.51, *p* = 0.26; no *trial* *×* *group* interaction *F*_(33,484)_ = 1.30, *p* = 0.13). The performance improvements leveled off at the end of day 1 such that PerfScore_all_ changed only minimally across the last 3 trials (<10% of the overall learning gains) even though statistics revealed a trend towards a significant trial main effect (*F*_(2,88)_ = 2.86, *p* = 0.06). There was no significant *trial* × *group* interaction (*F*_(6,88)_ = 0.31, *p* = 0.93) nor main group effect (*F*_(3,44)_ = 2.43, *p* = 0.08) indicating that the plateau effect was not significantly different across groups (Figure 1C).

Successful consolidation was tested by reactivating the motor memory on day 2 (tapping SeqLearn 10 times). This reactivation revealed further overnight changes when quantified via PerfScore_10_ which ranged between +12.1% ± 3.4 and +20.6% ± 1.8 (so called “offline gains”; main day effect *F*_(1,44)_ = 17.55, *p* < 0.001; Figures 2A,B). Even though PerfScore_10_ differed across groups (indicating that some subjects were better tappers than others, main *group* effect *F*_(3,44)_ = 3.06, *p* < 0.05), there was no significant *day* × *group* interaction (*F*_(3,44)_ = 0.95, *p* = 0.42) indicating that offline gains did not significantly differ across groups.

Central to our research question, we next tested whether the duration of the delay between reactivating SeqLearn and acquiring SeqInterf on day 2 had a significant influence on retention performance on day 3 (Figures 2A,B). We found a significant main *day* effect (*F*_(1,44)_ = 13.51, *p* < 0.001) and the subsequent *post hoc* analysis showed an overall decrease in performance from day 2 to day 3 (Tukey HSD *post hoc*, *p* < 0.001). We did not find a main *group* effect (*F*_(3,44)_ = 1.56, *p* = 0.21) while, most interestingly, we found a significant *day* × *group* interaction (*F*_(3,44)_ = 5.28, *p* < 0.01). A Tukey HSD *post hoc* analysis showed a significant drop in performance from day 2 to day 3 for the 20 s group (*p* < 0.01).

**Figure 3:**
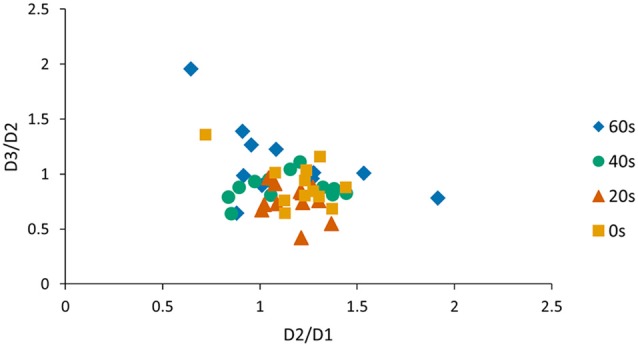
**Visualization of the correlation between D2/D1 and D3/D2 ratios.** We found a significant negative association between the D2/D1 ratio and the D3/D2 ratio when pooled across groups (*r* = −0.37, *p* < 0.01). As a consequence, the D2/D1 ratio was included as a covariate of no interest when analyzing the D3/D2 ratio.

One concern is that offline gains from day 1 to day 2 and performance changes observed from day 2 to day 3 are related. Therefore, we conducted an additional Pearson correlation analysis and found a significant negative association between the D2/D1 ratio and the D3/D2 ratio when pooled across groups (*r* = −0.37, *p* < 0.01; Figure 3) indicating that subjects who exhibited large offline gains from day 1 to day 2 tended to exhibit large losses in performance from day 2 to day 3. Since this association might have influenced our previous reconsolidation results we performed an additional control analysis and tested whether D3/D2 performance ratios differed across groups, even if the individual offline gain (i.e., D2/D1 ratio) was added as a covariate of no interest. Our model revealed a significant group *effect* (preplanned comparison *F*_(3,40)_ = 3.71, *p* = 0.03, one-sided) and a Tukey HSD *post hoc* analysis revealed a significant difference in the D3/D2 ratio for the 20 s group compared to the 60 s group (*p* < 0.01). This finding indicates that the delay significantly influenced memory deterioration due to reconsolidation, an effect that was found over and above individual difference in offline gains exhibited from D2 to D1.

Note, however, that performance was only degraded during the first trial on day 3 but increased quickly during the subsequent two tapping trials (Figure 2A, main *trial* effect *F*_(2,88)_ = 28.31, *p* < 0.001). This performance increase was not significantly different across groups (no *group* main effect *F*_(3,44)_ = 1.66, *p* = 0.19; no *trial* × *group* interaction *F*_(6,88)_ = 1.29, *p* = 0.27).

**Figure 4:**
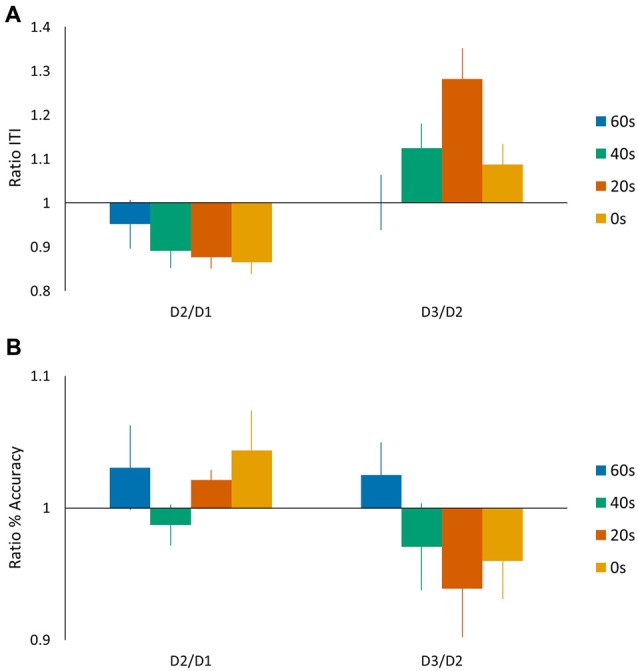
**Task performance represented by (A) speed (ITI (s)) and (B) accuracy (%) measures.** The speed and accuracy data are presented in a similar manner as shown in Figure 2B for the performance scores. Vertical bars indicate SEs.

In the above analyses we quantified tapping performance via a performance score based on a linear speed-accuracy function (de Beukelaar et al., 2014), whereas previous motor reconsolidation studies reported speed and accuracy measurements separately (Walker et al., 2003). We therefore repeated our main ANOVA analyses separately for the speed (ITI) and accuracy (%) measures (Figures 4A,B). For the speed measurement, we found a significant main *day* effect (*F*_(1,44)_ = 12.98, *p* < 0.001) and the subsequent *post hoc* analysis showed an overall decrease in performance from day 2 to day 3 (Tukey HSD *post hoc*, *p* < 0.001). We did not find a main *group* effect (*F*_(3,44)_ = 1.42, *p* = 0.25), while, most interestingly, we found a significant *day* × *group* interaction (*F*_(3,44)_ = 3.09, *p* < 0.05). A Tukey HSD *post hoc* analysis showed a significant drop in performance from day 2 to day 3 for the 20 s group (*p* < 0.01). For the accuracy measurement, we did not find any significant main effects (no main *day* effect *F*_(1,44)_ = 3.58, *p* = 0.07; no main *group* effect *F*_(3,44)_ = 0.50, *p* = 0.68) nor a *day* × *group* interaction (*F*_(3,44)_ = 1.48, *p* = 0.23). We also calculated the D2/D1 and D3/D2 ratios for both speed (ITI) and accuracy (%) measures. We submitted the D3/D2 ratios to an ANOVA analysis and included the D2/D1 ratio as a covariate of no interest. For these measures separately, we did not find main *group* effects (preplanned comparison ANOVA, Accuracy: no main *group* effect *F*_(3,44)_ = 2.63, *p* = 0.11; Speed: no main *group* effect *F*_(3,44)_ = 0.97, *p* = 0.33). These findings suggest that the differences in performance score observed on day 3 were mainly driven by changes in performance speed (longer ITI) since changes in accuracy were generally minimal (on average around 5%).

**Figure 5:**
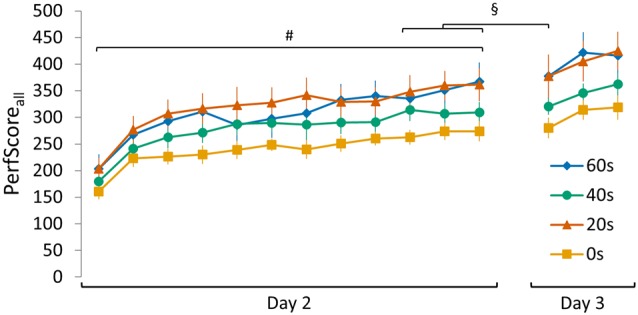
**Visualization of the SeqInterf PerfScore_all_ data of all experimental groups (0 s, 20 s, 40 s and 60 s).** A set of control analyses conducted on SeqInterf showed that: (i) initial PerfScore_all_ (i.e., performance on the first 30 s trial on day 2) did not differ between groups (no *group* main effect *F*_(3,44)_ = 1.092, *p* = 0.36); (ii) a clear increase in PerfScore_all_ over the course of training was evident (*trial* main effect *F*_(11,484)_ = 50.83, *p* < 0.001; no *group* main effect *F*_(3,44)_ = 2.32, *p* = 0.09; no *trial* × *group* interaction *F*_(33,484)_ = 0.94 *p* = 0.57); (iii) plateau PerfScore_all_ was not significantly different between groups (no *group* main effect *F*_(3,44)_ = 2.45, *p* = 0.08; no *trial* main effect *F*_(2,88)_ = 2.90, *p* = 0.06; no *trial* × *group* interaction *F*_(6,88)_ = 1.04, *p* = 0.41); (iv) and similar over-night improvements in performance were seen comparing the averaged PerfScore_all_ of the final three trials on day 2 with the retention PerfScore_all_ obtained during the first trial on day 3 for each group (main *day* effect *F*_(1,44)_ = 4.13, *p* < 0.05; no main *group* effect *F*_(3,44)_ = 2.52, *p* = 0.07; no *day* × *group* interaction *F*_(3,44)_ = 0.22, *p* = 0.88). Significant main *trial* effect is indicated by ^#^(*p* < 0.001). Significant main *day* effect is indicated by ^§^(*p* < 0.05). Vertical bars indicate SEs.

## Discussion

Here we explored the temporal dynamics of memory re-stabilization after reactivation, which represents an important experimental boundary condition for inducing motor memory degradation during reconsolidation. To do so, motor memory traces of a sequence tapping task were investigated using a well-established three-day design: on day 1 a novel motor memory was encoded, on day 2 the motor memory of the acquired sequence was reactivated and experimentally manipulated by learning an interfering sequence, and on day 3 the motor memory strength was retested (Walker et al., 2003; Censor et al., 2010; de Beukelaar et al., 2014; Hardwicke et al., 2016). We varied the duration of the delay between the brief reactivation of the previously acquired sequence and the interfering sequence in four experimental groups, so that in one group, the delay was 60 s, in the second group 40 s, in the third group 20 s and in the fourth group it was 0 s. Our results indicate that the duration between reactivation and interference critically influences the motor memory degradation process. These findings indicate that memory re-stabilization after reactivation is a dynamic process and that besides the length of reactivation also the delay between reactivation and interference constitutes a crucial boundary condition to test motor memory reconsolidation.

Subtle boundary conditions constrain whether a memory can be experimentally interfered with upon reactivation or not (Rodriguez-Ortiz and Bermúdez-Rattoni, 2007). While specific determinants of reconsolidation (e.g., age of the memory, intensity of training, reactivation length, and novelty of information provided during the reactivation session) have been identified in animal models (Milekic and Alberini, 2002; Eisenberg et al., 2003; Pedreira and Maldonado, 2003; Pedreira et al., 2004; Suzuki et al., 2004; Morris et al., 2006; Bustos et al., 2009; Wang et al., 2009; Auber et al., 2013; Díaz-Mataix et al., 2013), these remain less understood in humans (Schiller and Phelps, 2011; Auber et al., 2013; Sevenster et al., 2013). A previous study from our laboratory recently showed that the length of memory reactivation is a critical parameter when interfering with human motor memory reconsolidation (de Beukelaar et al., 2014). In particular, a short reactivation (less than 60 s) renders the memory labile and susceptible to degradation through interference, while a longer reactivation does not. Moreover, the results showed a relationship between the length of reactivation and motor memory degradation: the longer the reactivation phase, lower the decline in performance due to interference when retested 24 h later.

In the present study, subjects reactivated the motor memory by tapping 10 repetitions of the previously acquired sequence (lasting on an average for 14.0 s ± 2.3). Our previous study showed that this brief period of motor reactivation rendered the motor memory most susceptible to degradation due to interference (de Beukelaar et al., 2014). Here we replicated these previous findings by showing that a brief reactivation followed by an interfering task degrades motor memories when retested 24 h later. In this study, we further explored the influence of the duration (or rest period) between reactivation and interference in four experimental groups with delay durations of 0 s, 20 s, 40 s or 60 s. Interestingly, the duration of this rest period directly influenced the extent to which the memory could be degraded. This was indicated by two main findings: first, delays between 0 and 40 s resulted in average memory degradation, while a delay of 60 s resulted in memory conservation and even caused an average performance gain. When directly compared by an ANOVA we found a significant *day* × *group* effect which was driven by differences between the 20 s and 60 s group (significant *post hoc* test). We further showed that only the 20 s group exhibited a significant performance decrease from day 2 to day 3 while the performance decrease of other groups (0 s and 40 s) as well as the increase of the 60 s group did not reach significance. However, one has to note that the reactivation period was rather short (10 sequences = 50 finger taps) most likely resulting in only potentially small offline gains from day 2 to day 3. Thus, in summary, our statistical analysis revealed clear group differences whereby the time delay between reactivation and interference was the only experimental parameter that was varied. Second, we performed an additional control analysis and tested a statistical model that assumed a linear effect of the different delays (0 s, 20 s, 40 s and 60 s) on the performance changes from day 2 to day 3. This model was hypothesized *a priori* based on a separate study that used the same overall paradigm but manipulated the length of reactivation (de Beukelaar et al., 2014) rather than the delay between reactivation and interference. This linear model revealed a significant effect consistent with the interpretation that increasing delays caused a gradual change from performance degradation to performance conservation across groups. Together with the results of our previous study (de Beukelaar et al., 2014), these findings suggest that memory modification is regulated by two time-dependent processes: first, the memory is destabilized due to a brief reactivation (note that our results tentatively suggest that destabilization might have been more complete in the 20 s than in the 0 s group) which is then followed by re-stabilization requiring that sufficient time has passed before subjects are exposed to an interfering intervention. This effect is observed irrespective of whether subjects rest or practice the previously learned sequence during this “re-stabilization period”.

In accordance with de Beukelaar et al. (2014), performance of the sequence tapping task was quantified by calculating a linear speed-accuracy function; i.e., performance score. Since previous reconsolidation research using similar sequence tapping tasks often analyzed speed and accuracy measures separately (Walker et al., 2003), we also explored these measures in the current study. Taken together, our results indicate that interference was manifested as reduced speed (longer ITI), and to a lesser extent, reduced accuracy. These results are in line with previous findings since both parameters independently suggest that the current reconsolidation paradigm leads to degradation of the motor memory, however, specific parameters such as the length of the rest period influence the extent of degradation.

Overall, the results of the present study in combination with our previous work (de Beukelaar et al., 2014) support the *destabilization* theory, which states that that the reactivation of an existing memory leads to instability such that subsequent interference can induce memory loss or degradation (Nader et al., 2000; Walker et al., 2003; Kindt et al., 2009; Chan and LaPaglia, 2013). In both studies we showed that a short reactivation of an existing memory leads to instability of the memory and that interference early after reactivation (i.e., around 20 s) can induce degradation of the memory. When reactivation itself is prolonged by further practice or when the interfering intervention is presented outside the preferred time-window of destabilization (i.e., 14.0 s ± 2.3 tapping + 20 s rest), we show that limited or no degradation of the memory occurs. The most robust effect was found for the paradigms where short reactivations (≤30 s) were followed by interference after 20 s. It is worthwhile noting, however, that exact estimations of reactivation length or rest period are specific for the paradigm used in our studies, thus, the critical time-window for causing memory degradation via an inference approach is likely to differ across tasks and memory domains.

Although robust effects were found, we could not establish effective memory “deletion” without an additional fast recovery of performance when executed on day 3. Currently, it is not known whether interfering with reconsolidation causes a retrieval failure (retrieval theory) or an actual fractional erasure of the memory (storage theory; Tronson and Taylor, 2007). Importantly, previous motor reconsolidation research in animals (Peng and Li, 2009) and humans (Censor et al., 2010, 2014) indicate that interference only degrades but not effectively erases the formed motor memory. In human fear memory systems, however, a persistent erasure over a year has been established without relapse (Schiller et al., 2010; Björkstrand et al., 2015). It will be interesting for future researchers to investigate whether different protocols can potentially induce a more robust long-term drop in motor performance, for example, by more extensive interference learning, by repeating reactivation-interference sessions, or by applying other forms of interference (e.g., contextual interference).

To conclude, our data provide evidence that re-stabilizing motor sequence memories during reconsolidation does not necessarily require long periods of reactivation in order to be resistant to memory degradation, but that the availability of a specified rest period between a short reactivation and interference is sufficient. The effect of interference, shown as a drop in performance when retested 24 h later, was only short-lived which implies that reconsolidation interference results in subtle behavioral changes and requires a well-controlled experimental protocol taking into account all possible boundary conditions. Future studies should aim for a better understanding of the underlying memory dynamics of reconsolidation so that its potential as a therapeutic target in patients with memory disorders can be optimized.

## Author Contributions

TTdB designed the study; collected, analyzed and interpreted the data; drafted and revised the manuscript; gave final approval. DGW and NW designed the study; interpreted the data; revised the manuscript; gave final approval. KA and SPS interpreted the data; revised the manuscript; gave final approval.

## Conflict of Interest Statement

The authors declare that the research was conducted in the absence of any commercial or financial relationships that could be construed as a potential conflict of interest. The reviewers EG, AB and JD declared their shared affiliation, and the handling Editor states that the process nevertheless met the standards of a fair and objective review.
